# Negative perceptions toward older adults and life satisfaction among community-dwelling older citizens in Japan

**DOI:** 10.12688/f1000research.149132.2

**Published:** 2024-08-23

**Authors:** Yuho Shimizu, Kenichiro Sato, Susumu Ogawa, Daisuke Cho, Yoshifumi Takahashi, Daichi Yamashiro, Yan Li, Tomoya Takahashi, Keigo Hinakura, Ai Iizuka, Tomoki Furuya, Hiroyuki Suzuki

**Affiliations:** 1 Japan Society for the Promotion of Science, Tokyo, Japan; 2 The University of Tokyo, Tokyo, Japan; 3 Tokyo Metropolitan Institute for Geriatrics and Gerontology, Tokyo, Japan; 4 Aoyama Gakuin University, Tokyo, Japan

**Keywords:** Psychological Well-Being, Quality of Life, Stereotyping, Life Satisfaction, Multilevel Models

## Abstract

**Background:**

With the rapid aging of the population, increasing life satisfaction among older adults is essential. Negative perceptions of older adults are internalized, leading to poor mental health. This study hypothesized that participants with more negative perceptions of older adults would have lower life satisfaction.

**Methods:**

A cross-sectional survey of older adults was conducted across five wards and four cities in Tokyo, Japan. Participants responded to questions regarding demographics, life satisfaction, and negative perceptions of older adults. Data from 285 participants (264 women, *M* = 71.97 years) were analyzed.

**Results:**

The intraclass correlation coefficient for life satisfaction concerning residential areas was. 03 (95% confidence interval [CI] = [-.03, .10]). Instead of multilevel models, a multiple regression model with life satisfaction as the dependent variable and negative perceptions of older adults and demographics as the independent variables yielded the best fit. Results indicated that participants with more negative perceptions of older adults reported lower life satisfaction ( *β* = -.16, 95% CI = [-.28, -.04], *p* = .008), supporting our hypothesis.

**Conclusions:**

This study was constrained by limited variance in residential areas and a predominantly female participant pool. Previous studies have shown that higher life satisfaction is associated with increased social participation and extended life expectancy, and interventions aimed at enhancing life satisfaction in older adults are significant. Further exploration is warranted to ascertain whether a causal relationship exists, wherein more negative perceptions of older adults diminish life satisfaction.

## Introduction

Globally, the population is aging rapidly. This is especially evident in Japan, where 29.0% of the population will be 65 years or older by 2022. ^1^ While social problems associated with aging have become more apparent, older adults generally have a higher level of life satisfaction than other generations. ^2^^,^^3^ However, life satisfaction among older adults in Japan has declined in recent years. ^4^ As life satisfaction among older people is closely associated with a higher level of physical health, ^5^ increased social participation, ^6^ and longer life expectancy, ^7^^,^^8^ efforts to increase life satisfaction in this demographic are of great importance.

One variable that could be related to life satisfaction among them is negative perceptions toward older adults, which is how older citizens perceive the social group of “older adults.” Stereotype embodiment theory (SET) assumes that older adults internalize negative old-age perceptions. ^9^ Moreover, the SET argues that older adults who hold more negative perceptions are more likely to experience various negative effects. Previous studies have shown that older adults with more negative perceptions have poorer mental health, ^10^^,^^11^ lower cognitive function, ^12^ and slower recovery from illness. ^13^ Similar to the SET, the risks of ageism model ^14^ suggest that three factors broadly inhibit active aging: “stereotype embodiment,” “stereotype threat,” and “being a target of ageism.” Based on the above findings, negative perceptions toward older adults among the older participants will be associated with lower life satisfaction.

This study’s aim is examining the relationship between their negative perceptions toward older adults and life satisfaction. In this study, a cross-sectional survey of community-dwelling older adults in Tokyo, Japan, was conducted. The hypothesis is that participants with more negative perceptions toward older adults would have lower levels of life satisfaction. The study will also focus on the effect of the participant’s residential area, details of which are provided in the next section, along with information about the participant.

## Methods

### Participants

A paper-based questionnaire survey was used in this study. Participants read the questionnaire themselves and responded using a writing instrument. A power analysis assuming a small to moderate effect size ( *ρ* = .20, α = .05, 1– *β* = .80) yielded a required sample size of 193. Three hundred and two older Japanese individuals from Tokyo participated in this study. However, 17 participants were excluded from the analysis because they were < 65 years old. Thus, the data of 285 participants (aged 65–92, *M* = 71.97 years, *SD* = 5.16) were analyzed. The participants comprised 21 men and 264 women. They were older adults who voluntarily applied for a health course to train volunteers to read picture books to children, which was held in five wards and four cities in Tokyo from 2021–2022. In addition to population and economic size, each area had different main goals for the health course presented when recruiting participants (e.g., volunteer training and/or dementia prevention). Thus, an exploratory multilevel analysis was conducted to examine the hypothesis, considering the effect of participants’ residential areas. A summary of the participants by residential area is available in the Open Science Framework (OSF) repository ( https://doi.org/10.17605/OSF.IO/X6JSN). ^15^ Participants were required to attend the health course venue independently. There were no other exclusion criteria specifically, as long as the participants met the criteria of being 65 years of age or older. Note that the data for this study were collected before the implementation of the health course; therefore, results in this study do not include course effects. This study was approved by the first author’s institution for ethical review.

All procedures were in accordance with the ethical standards of Tokyo Metropolitan Institute for Geriatrics and Gerontology (approval number: 748; June 10, 2020) and with the 1964 Helsinki declaration and its later amendments or comparable ethical standards. Informed consent was obtained from all participants in written format.

### Measurements

Negative perceptions toward older adults were measured using the eight adjectives (five-point Likert scale). ^16^ Participants were asked the following question: “To what extent do you think older adults fit each of the following adjectives?” Adjectives presented to participants included “depressed” and “passive.” The mean was taken as the score (α = .89), with higher scores indicating more negative perceptions toward older adults.

Life satisfaction was measured using the five-items of the Satisfaction with Life Scale ^17^ measured on a seven-point Likert scale. Examples of items include: “In most ways, my life is close to my ideal.” The mean was taken as the score (α = .86), with higher scores indicating a higher level of life satisfaction. Demographic variables included years of education, age, and sex. Although there may be other confounding individual difference variables, other variables were not measured in this study in consideration of participants’ burden.

### Procedure and analysis

Participants received an explanation regarding the use of survey data for research purposes and agreed to participate in the study. Subsequently, they responded to demographic variables, life satisfaction, and negative perceptions toward older adults. The statistical software R (ver.4.1.0) was used for all analyses. The list of questions, data used in the analysis, scripts for R, histograms for each variable, and summary statistics can be accessed through the OSF.

## Results

The intraclass correlation coefficient (ICC) for life satisfaction concerning residential areas was.03 (95% CI = [-.03, .10]). Therefore, applying a multilevel analysis was not necessary. However, in this study, a simple multiple regression analysis (Model 1), a random effect with an analysis of covariance (RANCOVA) model, including the effect of residential area (Model 2), a random intercept and slope model with a group-level effect of negative perceptions toward older adults (Model 3), and a random intercept and slope model with a cross-level interaction (Model 4) were conducted. These models were compared in Table 1.

**Table 1.** Results for each model with life satisfaction as the dependent variable.

	Model 1		Model 2		Model 3		Model 4	
	*β*	95%CI	*β*	95%CI	*β*	95%CI	*β*	95%CI
NP	-.16 **	[-.28, -.04]	—		—		—	
NP (individual)	—		-.18 **	[-.30, -.05]	-.16	[-.33, .02]	-.15	[-.33, .04]
NP (area)	—		—		-.07	[-.58, .42]	-.08	[-.66, .44]
NP (ind.×area)	—		—		—		.02	[-.14, .18]
Education years	.06	[-.06, .18]	.06	[-.06, .18]	.06	[-.05, .19]	.06	[-.05, .19]
Age	-.07	[-.19, .06]	-.05	[-.17, .08]	-.03	[-.16, .09]	-.03	[-.16, .09]
Sex	-.09	[-.20, .03]	-.09	[-.20, .03]	-.09	[-.20, .03]	-.09	[-.20, .03]
AIC	808.53		826.30		831.20		836.38	
BIC	830.44		851.87		867.72		876.56	

*Note.* *β* is standardized. NP: Negative Perceptions; Sex: 0 = men, 1 = women; AIC: Akaike’s Information Criterion; BIC: Bayesian Information Criterion.

** *p* < .01.

Life satisfaction was used as the dependent variable in each analysis. In Model 1, a multiple regression analysis including the demographics (years of education, age, and sex) showed that participants with more negative perceptions toward older adults had lower life satisfaction ( *β* = -.16, 95% CI = [-.28, -.04], *p* = .008). In Model 2, a RANCOVA model with an individual-level effect (group-mean centering) of negative perceptions toward older adults was conducted and a similar effect to Model 1 ( *β* = -.18, 95% CI = [-.30, -.05], *p* = .005) was found. In Model 3, a random intercept and slope model, including a group-level effect (deviating from regional means) of the negative perceptions toward older adults, was employed. The results showed that the individual-level effect ( *β* = -.16, 95% CI = [-.33, .02], *p* = .11) and the group-level effect ( *β* = -.07, 95% CI = [-.58, .42], *p* = .80) were not significant. In Model 4, a random intercept and slope model, including cross-level interaction, was employed. The results showed that the individual-level effect ( *β* = -.15, 95% CI = [-.33, .04], *p* = .18), the group-level effect ( *β* = -.08, 95% CI = [-.66, .44], *p* = .77), and the cross-level interaction ( *β* = .02, 95% CI = [-.14, .18], *p* = .85) were not significant.

Model comparisons were conducted using the Akaike’s information criterion and the Bayesian information criterion, and Model 1 was determined to fit the data best. Note that when participants aged under 70 ( *n* = 103) and 70 or over ( *n* = 182) were analyzed separately; the results for both groups were similar to those in the main manuscript (see OSF). Similar results were obtained in a multiple regression analysis using a dummy variable for the residential area, with zero for the ward and one for the city (see OSF). Thus, the hypothesis in this study that participants with more negative perceptions toward older adults have lower levels of life satisfaction was supported.

## Discussion

In this study, a cross-sectional survey of community-dwelling older adults in nine areas in Tokyo was conducted to examine the relationship between negative perceptions toward older adults and life satisfaction. Each multilevel model fits the data worse than the simple multiple regression analysis. The results showed that participants with more negative perceptions toward older adults had lower life satisfaction, thus supporting the hypothesis. Note that a reverse relationship could also be assumed: individuals with lower life satisfaction have more negative perceptions toward older adults. The same association was found when this possibility was examined, as in the main manuscript (see OSF).

In this study, the ICC for life satisfaction in residential areas was small, and the fit of each multilevel model was relatively low. One reason for this may be that the participants were limited to those in a health course, training volunteers to read picture books. While detailed motivations for participating in the course varied from individual to individual, the attitude of “I am interested in picture books” and “I want to work as a volunteer” was probably shared by almost all participants. Thus, possibly, the group-level effect was relatively small due to the presence of factors common to participants across residential areas.

As this study is a cross-sectional survey, it should be examined whether a causal relationship exists between more negative perceptions toward older adults and decreased life satisfaction. Meanwhile, given that older adults with negative perceptions toward themselves have poorer mental health, ^10^^,^^11^ positively changing their perceptions toward older adults is important. One way to achieve this is to present people with information that contradicts negative old-age stereotypes. For example, negative stereotypes exist that “older adults are prone to illness,” but it was shown that presenting people with the content that “many older adults are healthy enough and able to live on their own” decreased ageism toward older adults. ^18^^,^^19^ Although these findings targeted younger people, a similar experimental manipulation for older adults may affirm their perceptions toward older adults. Future empirical studies are required to positively change the perceptions toward old age among older adults.

In this study, a cross-sectional survey of community-dwelling older adults who voluntarily applied for a health course to train volunteers to read picture books to children in Tokyo was conducted. The findings of this study can be utilized to improve programs aimed at promoting the health of older adults. For example, reducing negative perceptions toward older adults prior to enrolling in a program may make it easier for them to achieve greater life satisfaction. As noted above, this study is limited to the examination of correlations, and longitudinal experiments should be conducted in the future.

This study had two major limitations. First, the areas covered in this study were all located in Tokyo, Japan, and the regional differences were small. Compared with Japan’s underpopulated regions, all nine regions in this study share a high population density and a very small number of people engaged in agriculture, forestry, and fisheries. Therefore, follow-up studies are required to select regions with significantly different geopolitical characteristics from a wide range of prefectures. Second, the participation was skewed toward women. Since this study was conducted before a health course to train volunteers to read picture books to children, women comprised the majority of the participants. Note that results similar to those in the main manuscript were obtained when the analysis was limited to women’s data (see OSF); however, this study could not make adequate comparisons between men and women. Therefore, the findings in this study should be re-examined with a sufficient number of male participants.

In this study, a significant association between negative perceptions toward older adults and life satisfaction was found among community-dwelling older adults. Interventions that increase life satisfaction in older adults are meaningful because higher life satisfaction leads to increased social participation and longer life expectancies. Thus, it would be useful to focus on the negative perceptions toward older adults. The strength of this study is that the analysis of the relationship between negative perceptions toward older adults and life satisfaction takes into account the effects of residential areas. Future studies on life satisfaction and negative perceptions toward older adults in a broader geographic area should consider regional effects.

### Ethical considerations

All procedures were in accordance with the ethical standards of the research committee of Tokyo Metropolitan Institute for Geriatrics and Gerontology, (approval number: 748; June 10, 2020 and with the 1964 Helsinki declaration and its later amendments or comparable ethical standards. Written Informed consent was obtained from all individual participants included in the study.

## Data Availability

The data used in the analysis is available in the Open Science Framework (OSF) repository: Negative perceptions toward older adults and life satisfaction among community-dwelling older citizens in Japan, https://doi.org/10.17605/OSF.IO/X6JSN. ^15^ The project contains the following data:
• old image place4.csv (dataset), • old image place code_3.R (the R codes for analysis), •
OSF_supplemental_1.pdf (supplementary file of the manuscript). This data was collected and formed by the authors of this paper. The license of this data is CC-BY 4.0 International.
