# Supplementary material for: Negative perceptions toward older adults and life satisfaction among community-dwelling older citizens in Japan
Source: F1000Res. 2025 Mar 11;13:467. Originally published 2024 May 9. [Version 4] doi: 10.12688/f1000research.149132.4 (PMC11780261; doi:10.12688/f1000research.149132.4)
Supplement: Peer review history

# Reviewer report: Reviewer response for version 4

**DOI:** 10.5256/f1000research.178731.r370479

**Published:** 2025-04-04

**Authors:** Susiana Nugraha

**Affiliations:** 1 Public Health Department, Faculty of Health Science, Universitas Indonesia, University of Respati Indonesia, Jakarta, Special Region of Jakarta, Indonesia

Thank you for making significant improvements to the review given. All of reviewer comments have been revised accordingly. I have no other comment for this article.

 In my opinion, this article is very worthy of being accepted.

Is the work clearly and accurately presented and does it cite the current literature?

Partly

If applicable, is the statistical analysis and its interpretation appropriate?

Partly

Are all the source data underlying the results available to ensure full reproducibility?

Yes

Is the study design appropriate and is the work technically sound?

Yes

Are the conclusions drawn adequately supported by the results?

No

Are sufficient details of methods and analysis provided to allow replication by others?

Partly

Reviewer Expertise:

Gerontology, Public Health, Mental Health, Health Promotion

I confirm that I have read this submission and believe that I have an appropriate level of expertise to confirm that it is of an acceptable scientific standard.

# Reviewer report: Reviewer response for version 4

**DOI:** 10.5256/f1000research.178731.r370476

**Published:** 2025-04-04

**Authors:** Chang Hyun Seo

**Affiliations:** 1 University of Nevada Reno, Reno, USA

I have reviewed the revised version and the authors’ responses to my previous comments. The revisions appear appropriate and sufficiently address the concerns I raised.

 I would be happy to update my review status to Approved.

Is the work clearly and accurately presented and does it cite the current literature?

Partly

If applicable, is the statistical analysis and its interpretation appropriate?

Partly

Are all the source data underlying the results available to ensure full reproducibility?

Yes

Is the study design appropriate and is the work technically sound?

Partly

Are the conclusions drawn adequately supported by the results?

Partly

Are sufficient details of methods and analysis provided to allow replication by others?

Yes

Reviewer Expertise:

Social Policy analysis

I confirm that I have read this submission and believe that I have an appropriate level of expertise to confirm that it is of an acceptable scientific standard.

# Reviewer report: Reviewer response for version 4

**DOI:** 10.5256/f1000research.178731.r370477

**Published:** 2025-03-12

**Authors:** Joanne Hudson

**Affiliations:** 1 Swansea University, Swansea, Wales, UK

No further comments from me.

Is the work clearly and accurately presented and does it cite the current literature?

Yes

If applicable, is the statistical analysis and its interpretation appropriate?

I cannot comment. A qualified statistician is required.

Are all the source data underlying the results available to ensure full reproducibility?

No source data required

Is the study design appropriate and is the work technically sound?

Yes

Are the conclusions drawn adequately supported by the results?

Partly

Are sufficient details of methods and analysis provided to allow replication by others?

Yes

Reviewer Expertise:

Exercise Psychology; Older adults

I confirm that I have read this submission and believe that I have an appropriate level of expertise to confirm that it is of an acceptable scientific standard.

# Reviewer report: Reviewer response for version 3

**DOI:** 10.5256/f1000research.178089.r368208

**Published:** 2025-03-05

**Authors:** Joanne Hudson

**Affiliations:** 1 Swansea University, Swansea, Wales, UK

Thank you for making the changes to your manuscript. These have clarified the issues I raised.

Is the work clearly and accurately presented and does it cite the current literature?

Yes

If applicable, is the statistical analysis and its interpretation appropriate?

I cannot comment. A qualified statistician is required.

Are all the source data underlying the results available to ensure full reproducibility?

No source data required

Is the study design appropriate and is the work technically sound?

Yes

Are the conclusions drawn adequately supported by the results?

Partly

Are sufficient details of methods and analysis provided to allow replication by others?

Yes

Reviewer Expertise:

Exercise Psychology; Older adults

I confirm that I have read this submission and believe that I have an appropriate level of expertise to confirm that it is of an acceptable scientific standard.

# Reviewer report: Reviewer response for version 2

**DOI:** 10.5256/f1000research.169305.r360867

**Published:** 2025-02-11

**Authors:** Michiko Iwasaki

**Affiliations:** 1 Loyola University Maryland, Baltimore, USA

This study includes community-dwelling older adults who volunteer to support children's reading or take a dementia prevention course. Despite differing motivations, both groups engage in community-based services, justifying their treatment as one. A key limitation is the inclusion of 21 men. Given gender differences in health and ageism, focusing solely on women enhances internal validity while preserving a strong sample (n = 264). Clear reporting of effect sizes is essential, as the Beta coefficient for negative perception (0.18 or 0.16) on life satisfaction is small and should be interpreted accordingly. Lastly, the literature review on life satisfaction is weak, and I strongly recommend updating the introduction and discussion to emphasize older women.

Is the work clearly and accurately presented and does it cite the current literature?

Partly

If applicable, is the statistical analysis and its interpretation appropriate?

Partly

Are all the source data underlying the results available to ensure full reproducibility?

Yes

Is the study design appropriate and is the work technically sound?

Yes

Are the conclusions drawn adequately supported by the results?

Partly

Are sufficient details of methods and analysis provided to allow replication by others?

Yes

Reviewer Expertise:

Assessment of ageism; caregiving, and minority mental health

I confirm that I have read this submission and believe that I have an appropriate level of expertise to confirm that it is of an acceptable scientific standard, however I have significant reservations, as outlined above.

# Response

**Published:** 2025-02-26

**Authors:** Yuho Shimizu

Reply to Reviewer 5

 We express our appreciation to the reviewer for their insightful comments on our manuscript, which have helped us significantly improve the paper. Additionally, we have revised our manuscript according to the reviewer’s comments. Please find below our point-by-point responses to the comments.

 1) This study includes community-dwelling older adults who volunteer to support children's reading or take a dementia prevention course. Despite differing motivations, both groups engage in community-based services, justifying their treatment as one.

- We appreciate the reviewer’s comments. The same point was also raised by Reviewer 3.
- There were some other objectives for the course in addition to those listed in the original manuscript. Some areas had more than one of those objectives, while others had only one. In addition, the objectives of the course may have differed depending on the year of implementation. Therefore, in the past modification, we had added the following statement to the first paragraph of **Participants**: “ In addition to population and economic size, each area had different main goals for the health course presented when recruiting participants (e.g., volunteer training, dementia prevention, long-term care prevention, and/or social participation).”
- Participants knew the objective(s) of the course in advance. However, some areas had more than one of those objectives, while others had only one. In addition, as the course content remains largely the same between areas, the objectives of the course might not have a significant impact on the results (In fact, the effect of the area has not been seen). Moreover, it was unclear how strongly participants understood the course objectives. Based on the above, in the past modification, we had added the following statement to the first paragraph of **Participants**: “ Note that, some areas had more than one of those objectives, while others had only one. In addition, as the course content remains largely the same between areas, the objectives of the course might not have a significant impact on the results. Moreover, it was unclear how strongly participants understood the course objectives. Therefore, this study analyzed the effect of merely residential areas and did not focus on the differences in the objectives of the course.”

2) A key limitation is the inclusion of 21 men. Given gender differences in health and ageism, focusing solely on women enhances internal validity while preserving a strong sample ( *n* = 264).

- We thank the reviewer for your helpful comment regarding this point. As stated in the fifth paragraph of **Discussion**, we have already confirmed that the same results can be obtained by analyzing only women’s data. Please see the supplemental material on the Open Science Framework (OSF) repository.

3) Clear reporting of effect sizes is essential, as the Beta coefficient for negative perception (0.18 or 0.16) on life satisfaction is small and should be interpreted accordingly.

- We thank the reviewer for your helpful comment regarding this point. As you pointed out, the relationship between the variables is not strong enough. Accordingly, we have added the following statement to the first paragraph of **Discussion**: “ However, the association between negative perceptions toward seniors and life satisfaction is not strong enough, so we should be careful about over-interpreting the results.”

4) Lastly, the literature review on life satisfaction is weak, and I strongly recommend updating the introduction and discussion to emphasize older women.

- We thank the reviewer for this suggestion. We have added the following statement to the second paragraph of **Introduction**: “ There are other psychological variables that affect the life satisfaction of older people, although these are not examined in detail in this study. For a review of variables that contribute to life satisfaction among older adults in Asia, please refer to the following lite rature. ^15^ ”
- We have also added the following statement to the last paragraph of **Discussion**: “ In this study, we mainly analyzed data from older women, but we should also consider older men in the future.”

We have submitted the revised version of the manuscript. We look forward to hearing from you regarding our revised manuscript and would happily respond to any further questions and comments you may have.

# Reviewer report: Reviewer response for version 2

**DOI:** 10.5256/f1000research.169305.r360865

**Published:** 2025-02-07

**Authors:** Chang Hyun Seo

**Affiliations:** 1 University of Nevada Reno, Reno, USA

1. The title wording remains slightly ambiguous regarding the meaning of "negative perceptions toward older adults." Adding a clarification sentence in the Introduction would improve clarity.

 2. The sample is heavily skewed toward women (264 women, 21 men), which limits the generalizability of findings. A clearer discussion of this limitation is needed.

 3. The 95% confidence intervals (CIs) for the group-level and cross-level interaction effects in Models 3 and 4 are quite wide (e.g., [-0.66, 0.44] in Model 4), indicating a high level of uncertainty in these estimates. Discussing the implications of wide CIs in the limitations section would strengthen the manuscript.

 4. The claim that these findings can improve programs promoting the health of older adults appears somewhat speculative, as the study does not directly test interventions. I recommend softening this statement or rewording it to reflect the exploratory nature of the study. For example, instead of stating that the findings 'can improve programs,' the authors could clarify that the results may provide insights for designing future interventions.

Is the work clearly and accurately presented and does it cite the current literature?

Partly

If applicable, is the statistical analysis and its interpretation appropriate?

Partly

Are all the source data underlying the results available to ensure full reproducibility?

Yes

Is the study design appropriate and is the work technically sound?

Partly

Are the conclusions drawn adequately supported by the results?

Partly

Are sufficient details of methods and analysis provided to allow replication by others?

Yes

Reviewer Expertise:

Social Policy analysis

I confirm that I have read this submission and believe that I have an appropriate level of expertise to confirm that it is of an acceptable scientific standard, however I have significant reservations, as outlined above.

# Response

**Published:** 2025-02-09

**Authors:** Yuho Shimizu

Reply to Reviewer 4

 We express our appreciation to the reviewer for their insightful comments on our manuscript, which have helped us significantly improve the paper. Additionally, we have revised our manuscript according to the reviewer’s comments. Please find below our point-by-point responses to the comments.

**The title wording remains slightly ambiguous regarding the meaning of “negative perceptions toward older adults.” Adding a clarification sentence in the Introduction would improve clarity.**

- We thank the reviewer for this suggestion. We have added the following statement to the second paragraph of **Introduction**: “ …negative perceptions toward older adults, which is how older citizens perceive the social group of “older adults.” Some people view older adults in a positive light, while others view them in a negative light.”

** The sample is heavily skewed toward women (264 women, 21 men), which limits the generalizability of findings. A clearer discussion of this limitation is needed.**

- We thank the reviewer for this suggestion. The fact that the participants are biased toward women is already discussed in the second to last paragraph of **Discussion**. To elaborate on the content, we have added the following statement to the paragraph: “ It may be important to promote post-retirement social participation, especially with regard to men.”

**  The 95% confidence intervals (CIs) for the group-level and cross-level interaction effects in Models 3 and 4 are quite wide (e.g., [-0.66, 0.44] in Model 4), indicating a high level of uncertainty in these estimates. Discussing the implications of wide CIs in the limitations section would strengthen the manuscript.**

- We thank the reviewer’s helpful comment regarding this point. We have added the following statement to the second to last paragraph of **Discussion**: “ Third, 95% Cis for the group-level and cross-level interaction effects in Models 3 and 4 were wide. This indicates that the estimates contained large uncertainties. This may be due to insufficient sample size for each area. While it is significant that the survey was conducted in a wide range of areas, the number of participants in each area was insufficient. Therefore, elaborate estimates of group-level effects and interactions should be made in the future.”

** The claim that these findings can improve programs promoting the health of older adults appears somewhat speculative, as the study does not directly test interventions. I recommend softening this statement or rewording it to reflect the exploratory nature of the study. For example, instead of stating that the findings ‘can improve programs,’ the authors could clarify that the results may provide insights for designing future interventions.**

- We thank the reviewer’s helpful comment regarding this point. As course improvement is not the main objective of this study, we have added the following statement to the fourth paragraph of **Discussion**: “ Although course improvement is not the main objective of this study, the findings of this study can be partially utilized to improve programs aimed at promoting the health of older adults.”

We have attached the revised version of the manuscript. We look forward to hearing from you regarding our revised manuscript and would happily respond to any further questions and comments you may have.

# Reviewer report: Reviewer response for version 2

**DOI:** 10.5256/f1000research.169305.r359028

**Published:** 2025-01-29

**Authors:** Joanne Hudson

**Affiliations:** 1 Swansea University, Swansea, Wales, UK

(1) Exploring the role of residential area in the relationship under investigation is relevant but it would be helpful if some literature were included in the Introduction to provide a rationale for including residential area as a factor in the study.

 (2) Please identify which Wards and Cities were categorised as volunteering and dementia prevention in the supplemental material if this is relevant. 

 (3) Were the volunteers aware of the main goal for the health course (dementia prevention or volunteering)? If so, would this have influenced participant recruitment? 

 (4) I'm not quite clear on whether or not residential areas with different main goals for the health course were compared  - this is particularly important if volunteers were aware of these (see comment above). Please could you clarify this? 

 (5) Please review the following statement in the Discussion as the study was not aimed at finding out how to improve such programmes but at the relationships between individual difference factors in people attending them : "The findings of this study can be utilized to improve programs aimed at promoting the health of older adults." This comment is related to the answer "partly" to the final question above.

Is the work clearly and accurately presented and does it cite the current literature?

Yes

If applicable, is the statistical analysis and its interpretation appropriate?

I cannot comment. A qualified statistician is required.

Are all the source data underlying the results available to ensure full reproducibility?

No source data required

Is the study design appropriate and is the work technically sound?

Yes

Are the conclusions drawn adequately supported by the results?

Partly

Are sufficient details of methods and analysis provided to allow replication by others?

Yes

Reviewer Expertise:

Exercise Psychology; Older adults

I confirm that I have read this submission and believe that I have an appropriate level of expertise to confirm that it is of an acceptable scientific standard, however I have significant reservations, as outlined above.

# Response

**Published:** 2025-02-09

**Authors:** Yuho Shimizu

Reply to Reviewer 3

 We express our appreciation to the reviewer for their insightful comments on our manuscript, which have helped us significantly improve the paper. Additionally, we have revised our manuscript according to the reviewer’s comments. Please find below our point-by-point responses to the comments.

  

- Exploring the role of residential area in the relationship under investigation is relevant but it would be helpful if some literature were included in the Introduction to provide a rationale for including residential area as a factor in the study.
- **Response: **We appreciate the reviewer’s comments. We have added the following statement to the last paragraph of **Introduction**: “ In a large survey of residents in a wide range of Japanese cities, it has been reported that people in more urban areas have fewer close neighbors who greet each other. ^15^ Thus, results may vary depending on the participant’s residential area. Details regarding their residential area are provided in the next section.”

 

- Please identify which Wards and Cities were categorized as volunteering and dementia prevention in the supplemental material if this is relevant.
- **Response: **We thank the reviewer’s helpful comment regarding this point. There were some other objectives for the course in addition to those listed in the original manuscript. Some areas had more than one of those objectives, while others had only one. In addition, the objectives of the course may have differed depending on the year of implementation. Therefore, we have added the following statement to the first paragraph of **Participants**: “ In addition to population and economic size, each area had different main goals for the health course presented when recruiting participants (e.g., volunteer training, dementia prevention, long-term care prevention, and/or social participation).”

 

- Were the volunteers aware of the main goal for the health course (dementia prevention or volunteering)? If so, would this have influenced participant recruitment?
- **Response: **We thank the reviewer’s helpful comment regarding this point. We have added the following statement to the first paragraph of **Participants**: “ Participants were informed in advance of the objective(s) of the course.”

 

- I'm not quite clear on whether or not residential areas with different main goals for the health course were compared. This is particularly important if volunteers were aware of these (see comment above). Please could you clarify this?
- **Response: **We thank the reviewer for this suggestion. As indicated in the above response, participants knew the objective(s) of the course in advance. However, some areas had more than one of those objectives, while others had only one. In addition, as the course content remains largely the same between areas, the objectives of the course might not have a significant impact on the results (In fact, the effect of the area has not been seen). Moreover, it was unclear how strongly participants understood the course objectives. Based on the above, we have added the following statement to the first paragraph of **Participants**: “ Note that, some areas had more than one of those objectives, while others had only one. In addition, as the course content remains largely the same between areas, the objectives of the course might not have a significant impact on the results. Moreover, it was unclear how strongly participants understood the course objectives. Therefore, this study analyzed the effect of merely residential areas and did not focus on the differences in the objectives of the course.”

 

- Please review the following statement in the Discussion as the study was not aimed at finding out how to improve such programmes but at the relationships between individual difference factors in people attending them: “The findings of this study can be utilized to improve programs aimed at promoting the health of older adults.” This comment is related to the answer “partly” to the final question above.
- **Response: **We thank the reviewer for this suggestion. As course improvement is not the main objective of this study, we have added the following statement to the fourth paragraph of **Discussion**: “ Although course improvement is not the main objective of this study, the findings of this study can be partially utilized to improve programs aimed at promoting the health of older adults.”

We have attached the revised version of the manuscript. We look forward to hearing from you regarding our revised manuscript and would happily respond to any further questions and comments you may have.

# Reviewer report: Reviewer response for version 2

**DOI:** 10.5256/f1000research.169305.r316523

**Published:** 2024-08-28

**Authors:** Anna Zalewska

**Affiliations:** 1 Faculty of Health Sciences, University of Lomza, Lomza, Poland

I thank the authors for making corrections according to the comments. I have no more comments.

Is the work clearly and accurately presented and does it cite the current literature?

Partly

If applicable, is the statistical analysis and its interpretation appropriate?

Yes

Are all the source data underlying the results available to ensure full reproducibility?

Yes

Is the study design appropriate and is the work technically sound?

Partly

Are the conclusions drawn adequately supported by the results?

Partly

Are sufficient details of methods and analysis provided to allow replication by others?

Partly

Reviewer Expertise:

health sciences

I confirm that I have read this submission and believe that I have an appropriate level of expertise to confirm that it is of an acceptable scientific standard.

# Reviewer report: Reviewer response for version 1

**DOI:** 10.5256/f1000research.163548.r297567

**Published:** 2024-07-16

**Authors:** Susiana Nugraha

**Affiliations:** 1 Public Health Department, Faculty of Health Science, Universitas Indonesia, University of Respati Indonesia, Jakarta, Special Region of Jakarta, Indonesia

This paper discusses about the negative perception of being older adult is associated with life satisfaction. The title of this paper is little bit biased in wording. the term of "negative perception of older adult" tend to created ambiguous meaning for reader. I suggest you to add some sentence to avoid any ambiguity.

 The introduction part does not clearly highlight the important of this study, as well as the state of art of this study has not well described.

 The method section does not clearly explain the instrument being used and its measurement techniques. The role of other confounding variable might necessary to identify.

 I would like to see some discussion of the finding of the papers in relation to recent findings and development in active ageing program in Tokyo. The conclusion should concisely mention the finding as well as the implication of the study to the program and policy development.

Is the work clearly and accurately presented and does it cite the current literature?

Partly

If applicable, is the statistical analysis and its interpretation appropriate?

Partly

Are all the source data underlying the results available to ensure full reproducibility?

Yes

Is the study design appropriate and is the work technically sound?

Yes

Are the conclusions drawn adequately supported by the results?

No

Are sufficient details of methods and analysis provided to allow replication by others?

Partly

Reviewer Expertise:

Gerontology and Public Health

I confirm that I have read this submission and believe that I have an appropriate level of expertise to confirm that it is of an acceptable scientific standard, however I have significant reservations, as outlined above.

# Response

**Published:** 2024-07-17

**Authors:** Yuho Shimizu

Reply to Reviewer 2

 We express our appreciation to the reviewer for their insightful comments on our manuscript, which have helped us significantly improve the paper. Additionally, we have revised our manuscript according to the reviewer’s comments. Please find below our point-by-point responses to the comments.

 The title of this paper is little bit biased in wording. the term of “negative perception of older adults” tend to created ambiguous meaning for reader. I suggest you to add some sentence to avoid any ambiguity.

- We thank the reviewer for this point. We have revised the title of our paper.

The introduction part does not clearly highlight the important of this study, as well as the state of art of this study has not well described.

- We sincerely thank the reviewer for this point. We have added the study’s aim and strength to the third paragraph of **Introduction**: “ This study’s aim is examining the relationship between their negative perceptions toward older adults and life satisfaction. In this study, a cross-sectional survey of community-dwelling older adults in Tokyo, Japan, was conducted. The hypothesis is that participants with more negative perceptions toward older adults would have lower levels of life satisfaction. The study will also focus on the effect of the participant's residential area, details of which are provided in the next section, along with information about the participant.”

The method section does not clearly explain the instrument being used and its measurement techniques. The role of other confounding variable might necessary to identify.

- We thank the reviewer’s helpful comment regarding this point. We have added the following statement to the first paragraph of **Participants** section of **Methods**: “ A paper-based questionnaire survey was used in this study. Participants read the questionnaire themselves and responded using a writing instrument.” We have also added the following statement to the second paragraph of **Measurements** section of **Methods**: “ Although there may be other confounding individual difference variables, other variables were not measured in this study in consideration of participants’ burden.”

I would like to see some discussion of the finding of the papers in relation to recent findings and development in active ageing program in Tokyo. The conclusion should concisely mention the finding as well as the implication of the study to the program and policy development.

- We thank the reviewer’s helpful comment regarding this point. We have added the following statemen to the fourth paragraph of **Discussion**: “ In this study, a cross-sectional survey of community-dwelling older adults who voluntarily applied for a health course to train volunteers to read picture books to children in Tokyo was conducted. The findings of this study can be utilized to improve programs aimed at promoting the health of older adults. For example, reducing negative perceptions toward older adults prior to enrolling in a program may make it easier for them to achieve greater life satisfaction. As noted above, this study is limited to the examination of correlations, and longitudinal experiments should be conducted in the future.”

We have submitted the revised version of the manuscript. We look forward to hearing from you regarding our revised manuscript and would happily respond to any further questions and comments you may have.

# Reviewer report: Reviewer response for version 1

**DOI:** 10.5256/f1000research.163548.r297565

**Published:** 2024-07-05

**Authors:** Anna Zalewska

**Affiliations:** 1 Faculty of Health Sciences, University of Lomza, Lomza, Poland

Thank you for the opportunity to review this paper. This is a timely issue to explore. It is mostly clear paper, there is, however, issues that must be resolved before the study can be accepted for indexing. Several comments and suggestions for the authors.

 - In the abstract, instead of a hypothesis, the authors should state the aim.

 - In the abstract, authors should not describe limitations in their conclusions.

 - In the abstract, authors should not refer to other studies in their conclusions, but present a conclusion from their own research.

 - The introduction does not exhaust the topic.

 - There is no clearly defined aim of the study.

 - The last part of the introduction regarding the organization of research should not be included in it (it is part of the material and methods subsection).

 - Lack of clear inclusion and exclusion criteria.

 - The results lack an all-encompassing table, a flowchart for the selection of patients included with the step-by-step reasons for exclusion.

 - Can the authors provide the Cronbach’s alpha value for scales used?

 - In addition to the limitations, it is also worth describing the strengths of the study.

 - Research articles usually do not use the word „we”, „our” and regularly use passive verbs.

Is the work clearly and accurately presented and does it cite the current literature?

Partly

If applicable, is the statistical analysis and its interpretation appropriate?

Yes

Are all the source data underlying the results available to ensure full reproducibility?

Yes

Is the study design appropriate and is the work technically sound?

Partly

Are the conclusions drawn adequately supported by the results?

Partly

Are sufficient details of methods and analysis provided to allow replication by others?

Partly

Reviewer Expertise:

health sciences

I confirm that I have read this submission and believe that I have an appropriate level of expertise to confirm that it is of an acceptable scientific standard, however I have significant reservations, as outlined above.

# Response

**Published:** 2024-07-17

**Authors:** Yuho Shimizu

Reply to Reviewer 1

 We express our appreciation to the reviewer for their insightful comments on our manuscript, which have helped us significantly improve the paper. Additionally, we have revised our manuscript according to the reviewer’s comments. Please find below our point-by-point responses to the comments.

 In the abstract, instead of a hypothesis, the authors should state the aim. In the abstract, authors should not describe limitations in their conclusions. In the abstract, authors should not refer to other studies in their conclusions, but present a conclusion from their own research.

- We appreciate the reviewer’s comments. Along with the reviewer’s comments, we have revised the **Abstract**.

The introduction does not exhaust the topic. There is no clearly defined aim of the study.

- We thank the reviewer’s helpful comment regarding this point. We have added the following statement to the third paragraph of **Introduction**: “ This study’s aim is examining the relationship between their negative perceptions toward older adults and life satisfaction.”

The last part of the introduction

 Regarding the organization of research should not be included in it (it is part of the material and methods subsection).

- We thank the reviewer’s helpful comment regarding this point. We have added the following statement to the first paragraph of **Methods**: “ In addition to population and economic size, each area had different main goals for the health course presented when recruiting participants (e.g., volunteer training and/or dementia prevention). Thus, an exploratory multilevel analysis was conducted to examine the hypothesis, considering the effect of participants’ residential areas.”

Lack of clear inclusion and exclusion criteria. The results lack an all-encompassing table, a flowchart for the selection of patients included with the step-by-step reasons for exclusion.

- We thank the reviewer for this suggestion. In this study, participants were older adults who voluntarily applied for a health course to train volunteers to read picture books to children. There were no other exclusion criteria specifically, as long as the participants met the criteria of being 65 years of age or older. Therefore, we have determined that the flowchart regarding participant exclusions is unnecessary. Accordingly, we have added the following statement to the first paragraph of **Methods**: “ Participants were required to attend the health course venue independently. There were no other exclusion criteria specifically, as long as the participants met the criteria of being 65 years of age or older.”

Can the authors provide the Cronbach’s alpha value for scales used?

- We thank the reviewer for this suggestion. We have added the Cronbach’ alpha for each scale.

In addition to the limitations, it is also worth describing the strengths of the study.

- We thank the reviewer’s helpful comment regarding this point. We have added the following statement to the last paragraph of **Discussion**: “ The strength of this study is that the analysis of the relationship between negative perceptions toward older adults and life satisfaction takes into account the effects of residential areas. Future studies on life satisfaction and negative perceptions toward older adults in a broader geographic area should consider regional effects.”

Research articles usually do not use the word “we”, “our” and regularly use passive verbs.

- We thank the reviewer for this point. The text has been corrected to a form that does not use “we” or “our.”

We have submitted the revised version of the manuscript. We look forward to hearing from you regarding our revised manuscript and would happily respond to any further questions and comments you may have.
